# *In memoriam –* David Ernest Minnikin (1939– 2021)

**DOI:** 10.1099/ijsem.0.005093

**Published:** 2021-11-05

**Authors:** Mike Goodfellow, Iain C. Sutcliffe

**Affiliations:** ^1^​ School of Natural and Environmental Sciences, Newcastle University, Newcastle upon Tyne NE1 7RU, UK; ^2^​ Faculty of Health and Life Sciences, Northumbria University, Newcastle upon Tyne NE1 8ST, UK


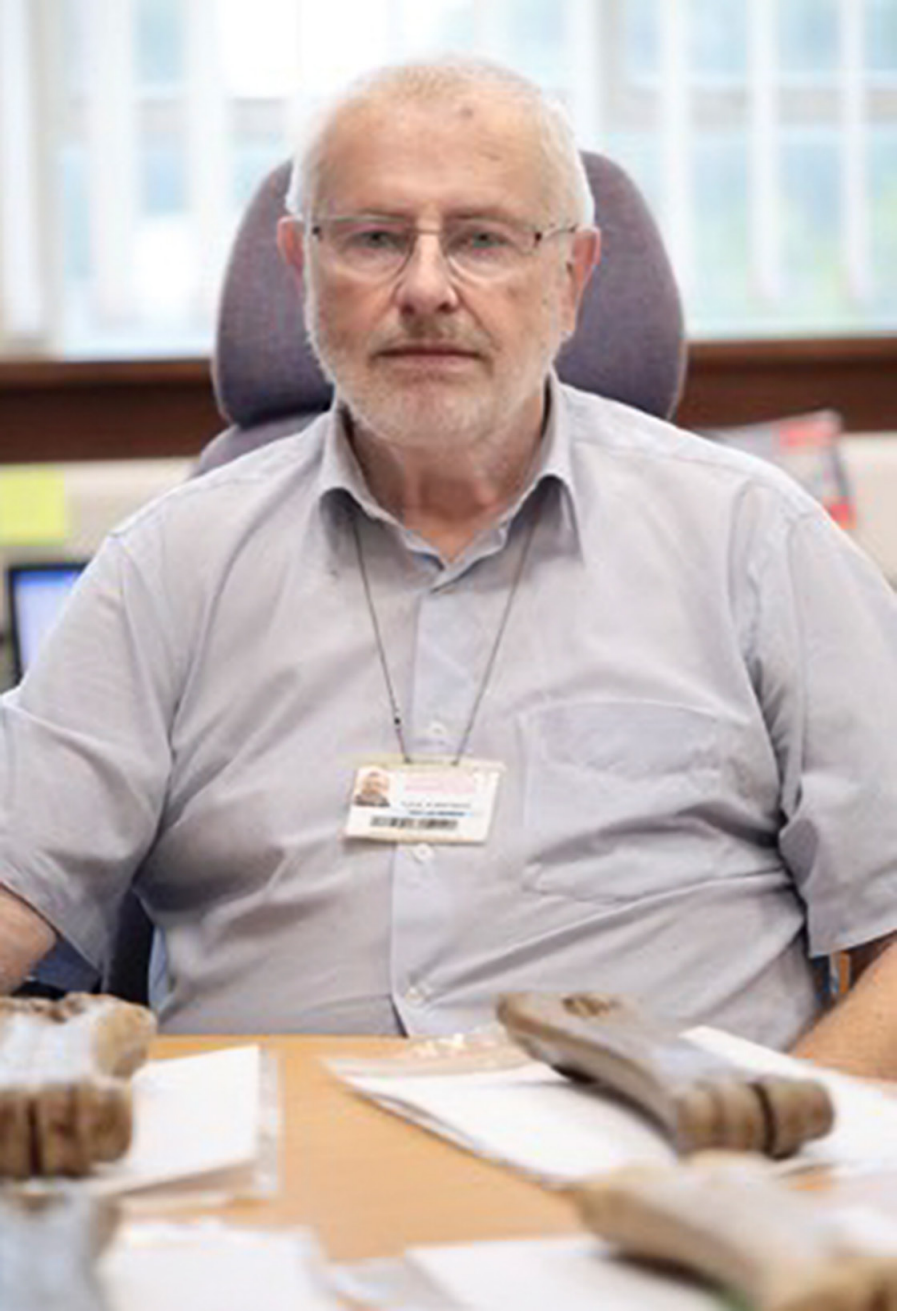


Members of the microbiological community across the world who knew David Minnikin personally or by reputation will be saddened to learn that he died on 20 July 2021 following a short illness.

Dave hailed from Newton, a small village in Northumberland, in the North East of England, where his parents were an integral part of a tightly knit farming community. Dave’s father was killed in Italy in the Second World War and this is why he was awarded a war widow’s scholarship to attend Lord Wandsworth, a boarding school in Hampshire. There, Dave excelled academically and athletically, notably at cricket, and won a scholarship to the University of Oxford. He was awarded a Masters in Chemistry and then stayed on to complete a D.Phil. in the Dyson Perrins Laboratory on the lipids of tubercle bacilli under the supervision of Nicholas Polgar. In 1967, he returned to his North Eastern roots to take up a position as Research Officer in Jim Baddiley’s Microbiological Chemistry Research Laboratory in the School of Chemistry at Newcastle University, and remained there for 35 years.

Dave made many outstanding contributions to microbial lipid chemistry in his career and is rightly recognized as one of the pioneers of microbial chemotaxonomy. He quickly gained an international reputation when the application of his experimental protocols led to dramatic improvements in the identification of pathogenic mycobacteria, thus helping improve the treatment of patients infected with ‘atypical mycobacteria’. The extension of these studies to other actinomycetes, notably those containing mycolic acids, provided an invaluable framework for the recognition of new pathogens and commercially significant actinomycetes. He and his taxonomic co-workers were the first to demonstrate that corynebacteria and related coryneform bacteria were *bona fide* actinomycetes. The significance of this work became clear when it was shown that the discontinuous distribution of chemical markers correlated with taxa defined using numerical and traditional taxonomic methods, thereby establishing chemotaxonomy as one of central planks of ‘polyphasic’ taxonomy. It is befitting that one of the key papers written by Dave and his colleagues in 1984 – ‘An integrated procedure for the extraction of bacterial isoprenoid quinones and polar lipids’ – has been cited nearly 4000 times.

Dave’s scientific interests spread well beyond chemotaxonomy, as witnessed by his work on the biosynthesis and structural analyses of complex lipids such as acyl-trehaloses, phenolic glycolipids, mycolic acids and phthiocerol dimycocerosate waxes. His understanding of the biological significance and chemical properties of complex mycobacterial lipids paved the way for his model of the cell envelope of *Mycobacterium tuberculosis *, presented at an international conference held at Bradford University in 1982. The ‘Minnikin model’ was published later that year in ‘The Biology of the Mycobacteria’ and still underpins our contemporary understanding of the organization of the cell envelopes of the hugely important pathogenic mycobacteria and related mycolic acid containing actinomycetes, in which the mycolic acids are now recognised to form an outer ‘mycomembrane’.

Dave was an ever-present and popular figure at international conferences on the biology of actinomycete bacteria where he was affectionately known amongst his friends as ‘minniquinones’. His original contributions to these meetings drew acclaim from leading lights in the actinomycete community such as Reiner Kroppenstedt (Germany), Romano Locci (Italy), Marian Mordarski (Poland), Jinsheng Ruan (China) and Larry Wayne (USA), as well as from key figures closer to home including Tony Jenkins, Colin Ratledge, John Stanford and Stan Williams. A succession of guest scientists came to Newcastle to be introduced to Dave’s chemotaxonomic procedures before returning home to spread the word; notable individuals included Mary Barton (Australia), Jean-Jacques Sanglier (Switzerland), Klaus Schaal (Germany) and Zhiheng Liu (China).

Dave demonstrated many other talents during his exceptional service to Newcastle University. His easy going, kind and caring demeanour were much appreciated by all whom he taught, especially by a steady flow of M.Sc. and Ph.D. students. Many of those he supervised followed his lead and went on to have successful careers in academia and biotechnology; they include Grace Alderson (UK), Gurdyal (Del) Besra (UK), Jongsik Chun (Korea), Martin Embley (UK), Mohamed Hamid (Sudan), Gilson Manfio (Brazil), Gerry Saddler (UK), Nevzat Sahin (Turkey) and Martha Trujillo (Spain). Dave was appointed as a Senior Research Officer in 1969 and somewhat belatedly was promoted to Senior Lecturer in 1985 and to a Personal Professorship in Microbial Chemistry in 1995, a position he held until 2002. Other contributions to the University community included spells as manager of the Analytical Services Unit in Chemistry, Treasurer of the Association of University Teachers and last, but not least, as a successful all-rounder in the University Staff Cricket Team. However, in many respects he preferred to play for Newton Cricket Club where he was renowned for his slow, non-turning offbreaks (‘tweakers’) and rustic bucolic batting.

In 2002, Dave and his joint research group with Del Besra moved to the University of Birmingham where he had more time to pursue his research interests, especially in the paleopathology of leprosy and tuberculosis. Although he regarded his interest in paleopathology as a hobby, he and his collaborators were able to show how mycolic acids extracted from archaeological specimens could be used to corroborate the insights being gained from ancient DNA studies, such as the recognition that human infections with *M. tuberculosis* predated those due to *Mycobacterium bovis *, reversing previous assumptions. Recently, he and his colleagues found evidence for tuberculosis in Neanderthals, a discovery which has implications not only for the evolution of the disease but also for that of the Pleistocene megafauna and humankind. Dave remained research-active to the end leaving behind several partially completed research papers.

Dave was the first of his family to benefit from higher education. He was part of a UK post-war generation encouraged to realise their talents in the collective interest. He responded to the opportunities that came his way and went on to achieve remarkably high standards in all that he did. Somehow, he found time to enjoy interests outside work and together with his wife Megan, a fellow organic chemist he met in Newcastle, he enjoyed gardening, travel and fell walking, notably climbing all ‘the Munros’ (mountains in Scotland that are over 3000 feet high). In addition, Dave was always ready for a game of cricket and sought time to socialise with friends over a few pints of real ale. He was devoted to Megan and went to super-human lengths to get pioneering drugs to combat the cancer that eventually claimed her life. In later years he was able to enjoy life with Marion, his second wife, a kindred spirit who shared his interests. Fortunately, they were able to have many happy years together before she pre-deceased him by a few months.

Dave was an open, but private individual, who carried his scientific talents with ease and humility. He was competitive in a quiet way even to the extent of trying to get a cheaper hotel room than his colleagues at conferences. On one famous occasion he turned up worse for wear on the first day of a conference in Antwerp, as he had been kept up all night following a police raid on a hotel that had offered remarkably cheap rates. Dave will be remembered for his many talents, his integrity, his engaging quirky traits and for his comradeship, self-effacing humour and for his humanity, especially by those whose careers were intertwined with his.

Dave is survived by his son Peter from his marriage to Megan and by wider family members who live in his beloved Newton where his remarkable life began. At heart he never really left Newton despite his Oxford education and scientific achievements.

